# GOT2: a moonlighting enzyme at the crossroads of cancer metabolism and theranostics

**DOI:** 10.3389/fimmu.2025.1626914

**Published:** 2025-08-06

**Authors:** Junxi Hu, Qingwen Liu, Qinglin Ren, Wenbo He, Jiaqi Hou, Xiaolin Wang, Yusheng Shu

**Affiliations:** ^1^ Clinical Medical College, Yangzhou University, Yangzhou, China; ^2^ Department of Graduate School, Dalian Medical University, Dalian, Liaoning, China; ^3^ Department of Thoracic Surgery, Northern Jiangsu People’s Hospital, Yangzhou, China

**Keywords:** moonlighting enzymes, GOT2, cancer metabolism, tumor microenvironment, immunotherapy

## Abstract

Moonlighting enzymes perform multiple distinct functions under different conditions without relying on gene fusion, splicing, or polymerization. Many classical metabolic enzymes, beyond their involvement in pathways like glycolysis and glutamine metabolism, also function as transcription factors, RNA-binding proteins, or signaling molecules. These dual roles are crucial in processes such as cancer metabolic reprogramming, immune evasion, and drug resistance. Glutamate oxaloacetate transaminase 2 (GOT2), a key example, is located in the mitochondria and catalyzes the transamination of aspartate and glutamate. Apart from its metabolic function, GOT2 also influences nuclear fatty acid metabolism and immune-related gene expression, affecting the tumor microenvironment. By integrating metabolic and signaling roles, GOT2 supports tumor cell adaptation to stress, promoting growth, survival, and immune escape. This multifunctionality positions GOT2 as a potential target for cancer diagnosis and therapy. This review discusses GOT2’s moonlighting roles and its clinical potential.

## 1 Introduction

**Figure 1:**
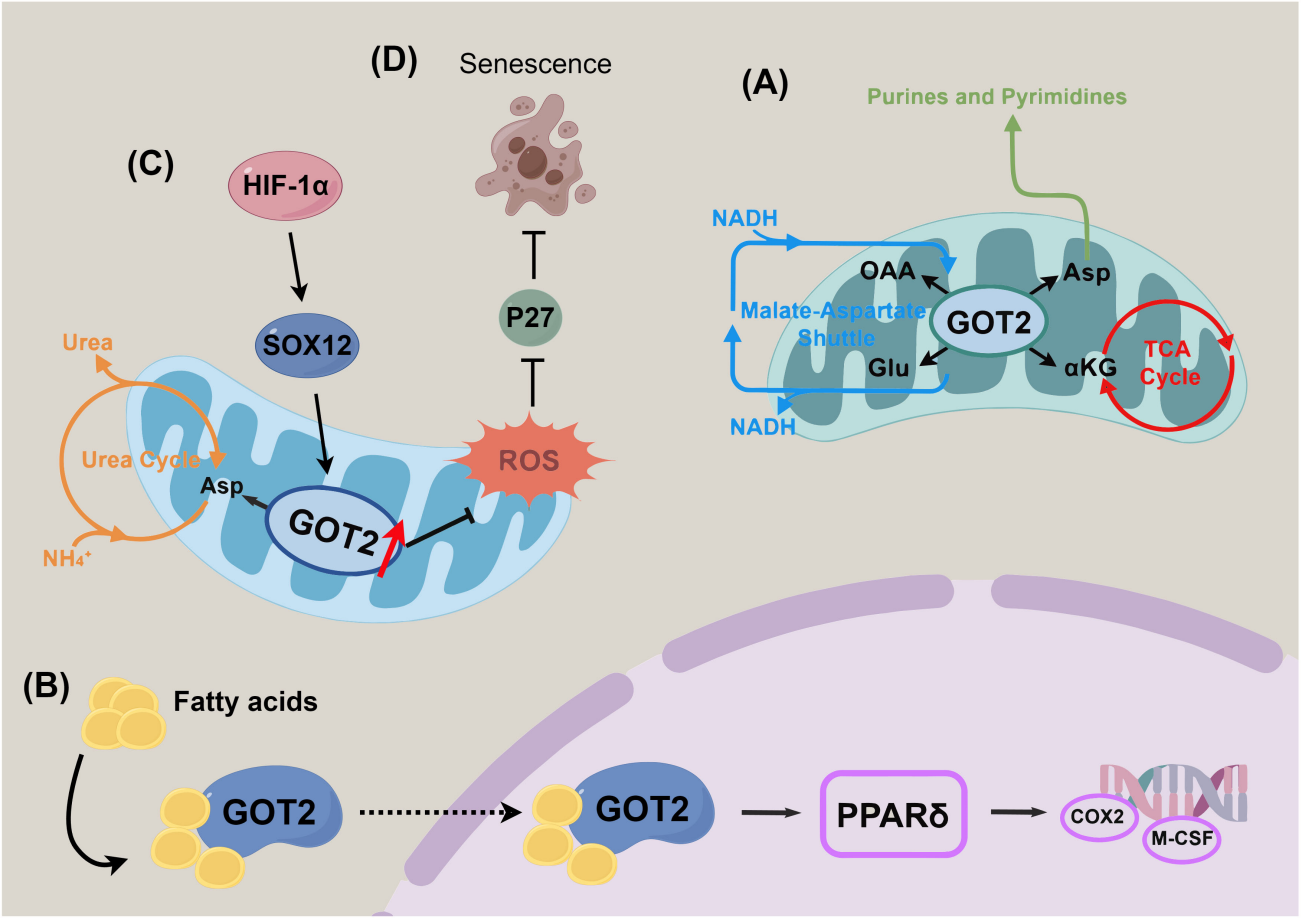
Schematic overview of the moonlighting functions of GOT2 in tumor biology. **(A)** Canonical metabolic roles of GOT2 in cellular energy production and redox balance. **(B)** Non-classical fatty acid-binding and membrane-associated activities contributing to cellular signaling and membrane dynamics. **(C)** Regulation of nitrogen homeostasis and metabolic reprogramming in response to tumor microenvironmental cues. **(D)** Involvement in cellular senescence and maintenance of antioxidant defense mechanisms.

Moonlighting enzymes refer to single polypeptide chains that perform two or more distinct functions under different microenvironmental conditions through conformational changes or subcellular localization shifts. This phenomenon does not rely on gene fusion, alternative splicing, or protein multimerization mechanisms (1, 2). Recent studies have increasingly shown that many classical metabolic enzymes are not limited to their traditional roles, such as glycolysis or glutamine metabolism, but also possess non-classical functions like transcriptional regulation, RNA binding, and signal transduction. These functions play crucial roles in tumor metabolic reprogramming, immune evasion, and drug resistance, challenging the traditional concept of “one enzyme, one function” (3–6). GOT2, a classical mitochondrial metabolic enzyme, has recently become a major research focus in the field of moonlighting enzymes due to its significant temporal and spatial dynamic regulation characteristics in tumor microenvironments and metabolic remodeling processes (7–10). GOT2 plays a particularly notable moonlighting role during malignant tumor progression. It regulates tumor cell lipid metabolism and immune-related gene expression by altering its subcellular localization, shaping the tumor microenvironment. Additionally, GOT2 integrates metabolic and signaling pathways, enabling tumor cells to adapt to stress conditions and facilitating their proliferation, survival, and immune escape (11–13). This review systematically summarizes the molecular mechanisms underlying the moonlighting functions of GOT2 (**Figure 1**), provides an in-depth analysis of interactions between metabolic networks and signaling pathways mediated by GOT2 during tumor progression (**Table 1**), and discusses the potential application prospects of its functional plasticity in developing targeted cancer therapies.

**Table 1:** Tumor-type-based classification highlighting GOT2 regulation differences.

Tumor type	Regulatory mechanism	Key polecules/Signaling pathways	GOT2 regulatory effect	Refs.
Breast Cancer	Transcriptional repression	BRCA1/ZBRK1	Loss of BRCA1 leads to GOT2 upregulation	(50)
Diffuse Large B-cell Lymphoma	Transcriptional activation	STAT3/NF-κB → c-Myc	Promotes GOT2 transcription	(51)
Colorectal Cancer	Transcriptional activation	SOX12 or SOX9 → c-Myc	Activates GOT2 and other metabolic enzymes	(34, 37)
Clear Cell Renal Cell Carcinoma	DNA methylation	Promoter hypermethylation, CpG site methylation	Downregulates GOT2 expression	(43, 52)
Pheochromocytoma	DNA methylation	TCA cycle mutations → abnormal DNMT activity	Upregulates GOT2 expression	(53)
Non-Small Cell Lung Cancer	Non-coding RNA regulation	circRNA/miRNA/postoperative microenvironment	Stabilizes or represses GOT2 expression	(55–57)
Pancreatic Cancer	Post-translational acetylation	Forms complex with MDH2, activates MAS	Maintains NAD^+^ balance, enhances metabolism	(58)
Prostate Cancer	Post-translational succinylation	Induced by ω-3 polyunsaturated fatty acids	Inhibits GOT2 function	(60)

## 2 Genetic features and mutations of GOT2

The human GOT2 gene is located on chromosome 16q21 (chr16:58,526,874–58,561,638), spans approximately 34.7 kb, and consists of 10 exons and 9 introns (14). It encodes a 430-amino-acid mitochondrial aspartate aminotransferase, with exon 2 at the 5’ end responsible for the mitochondrial targeting sequence (MTS) and exons 4–6 forming the highly conserved PLP (Pyridoxal 5’-phosphate)-dependent catalytic core. The gene exhibits strong evolutionary conservation among vertebrates. Although multiple rare nonsynonymous variants have been identified in population databases such as gnomAD, pathogenic mutations are predominantly observed in homozygous or compound heterozygous states and mainly affect key residues in the PLP-binding sites (e.g., Lys259, Arg387) or the dimer interface (e.g., Leu146, Phe322, Arg262) (15–17). Such mutations commonly result in severe loss of GOT2 enzymatic activity, disruption of aspartate shuttling, developmental delay, and epilepsy, with their deleterious mechanisms validated by genetic, biochemical, and structural studies (9, 18).

## 3 Structural dynamics and functional versatility of GOT2

**Figure 2:**
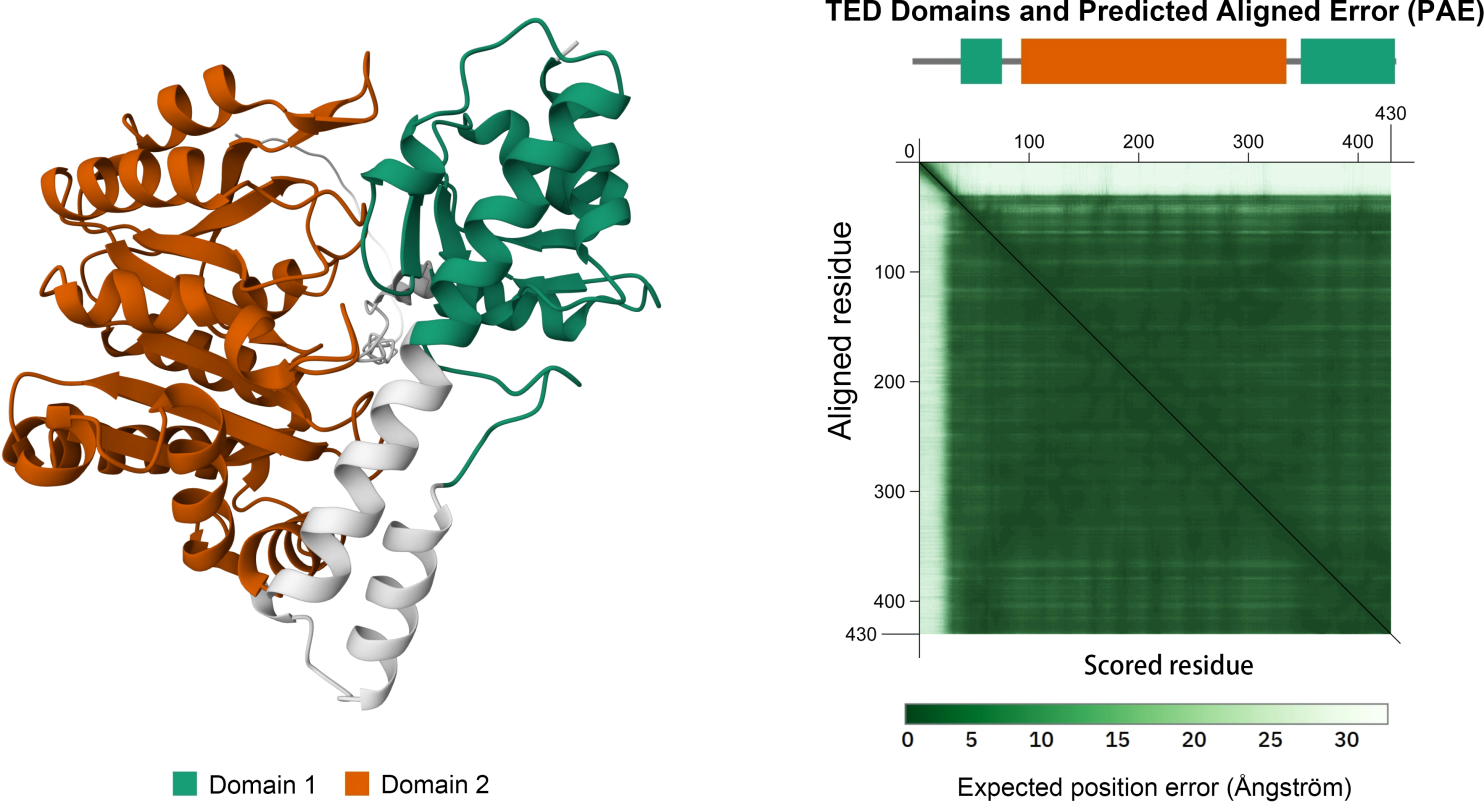
Domain architecture of the human GOT2 protein. The orange region (Hex: #BF5700) denotes the PLP-dependent aminotransferase domain responsible for its catalytic activity. Structure obtained from the AlphaFold Protein Structure Database (UniProt: P00505).

As shown in **Figure 2**, the yellow domain represents a typical type I PLP-dependent aspartate aminotransferase-like major domain. This domain is essential for maintaining transaminase activity by stabilizing the active site and facilitating the binding of the coenzyme PLP. The active site of GOT2 is deeply embedded within the PLP-binding pocket at the homodimer interface. Lys259 forms a Schiff base (internal aldimine bond) with PLP, while conserved residues such as His260, Trp261, and Ser262 collaboratively stabilize its spatial orientation and electronic environment. Arg387 and Asp223 further anchor the PLP phosphate group through salt bridges and hydrogen bonds, shaping the local proton-transfer microenvironment. During the catalytic cycle, the substrate aspartate is recognized by residues including Glu177, Arg246, and Thr130 upon entering the active site (17, 19). Its α-amino group undergoes transamination with PLP, forming an external aldimine intermediate. In this process, electrons are transiently delocalized through a polarized network toward His260 and Arg387, enabling amino group transfer and cleavage of the covalent bond, ultimately generating α-ketoglutarate and glutamate. Conformational changes involving the gating helix (residues 168–176) and substrate recognition loops regulate substrate entry and product release. Dynamic adjustments in the volume and polarity of the active pocket ensure substrate specificity. GOT2 functions as a homodimer, with dimerization stabilized by interface residues such as Leu146, Phe322, and Arg387. These residues are critical for maintaining the structural integrity of the catalytic core and the proper opening of the substrate channel. Disruption of the dimer interface—such as by Leu146Pro or Phe322Ser mutations—leads to exposure of the active site, impaired PLP binding, and near-complete loss of catalytic activity, ultimately resulting in disturbances in energy and amino acid metabolism. These molecular mechanisms have been validated through X-ray crystallography and site-directed mutagenesis studies (17, 20).

## 4 Canonical metabolic functions of GOT2

GOT2 is a key mitochondrial aminotransferase that links amino acid metabolism to central carbon flux and redox balance in both normal and malignant cells. By catalyzing the transamination of glutamate and oxaloacetate to produce aspartate and α-ketoglutarate, GOT2 ensures the availability of aspartate—essential for nucleotide biosynthesis and tumor cell proliferation—and supports the tricarboxylic acid (TCA) cycle via α-ketoglutarate anaplerosis, thereby sustaining mitochondrial energy metabolism (21–26). As a key component of the malate–aspartate shuttle (MAS), GOT2 facilitates the transfer of reducing equivalents from cytosolic NADH into the mitochondrial matrix, maintaining the NAD^+^/NADH balance required for oxidative phosphorylation and redox homeostasis (12, 22, 27–29). Disruption of GOT2 or MAS results in redox imbalance and impaired metabolic flux, which may cause neurological deficits or constrain tumor growth due to aspartate depletion and elevated oxidative stress (18, 30, 31). Beyond cancer, GOT2 activity is also critical for cellular differentiation and viability in multiple normal tissues, underscoring its fundamental role in maintaining physiological homeostasis (32, 33).

## 5 Moonlighting functions of GOT2 in cancer

### 5.1 Fatty acid-binding and membrane-associated functions

Besides its mitochondrial role, GOT2 has been identified as a plasma membrane-associated fatty acid-binding protein (FABPpm). Under conditions of cellular stress, organ injury, or lipid accumulation, GOT2/FABPpm regulates fatty acid oxidation and energy metabolism by mediating transmembrane transport of free fatty acids (34). While the role of GOT2 in fatty acid transport is well-established in normal tissues, its biological function in cancer has only recently been uncovered. In pancreatic ductal adenocarcinoma models, Abrego et al. found that GOT2 predominantly localizes to the nucleus, enhancing the transport of fatty acids such as arachidonic acid, thereby facilitating their binding to peroxisome proliferator-activated receptor δ (PPARδ) and significantly activating the PPARδ signaling pathway (35). These findings reveal a novel mechanism by which GOT2 drives tumor progression through regulating fatty acid oxidation and lipid signaling pathways, especially under conditions of stress or metabolic dysregulation (**Table 2**).

**Table 2:** Moonlighting functions of GOT2 and metabolites.

Moonlighting functions	Mechanism	Cancer type	Refs.
Regulates fatty acid oxidation	Interacts with PA and OA fatty acids	Primary hepatocytes	(35)
Fatty acid transport	Binds to PPARδ	Pancreatic ductal adenocarcinoma	(36)
Nitrogen balance regulation and urea cycle metabolism	Interacts with HIF-1α-SOX12 signaling axis	Colorectal cancer	(37)
Cellular senescence	Interacts with P27;ROS	Pancreatic ductal adenocarcinoma	(38)
Maintains NAD^+^ balance	Interacts with MDH2, activates MAS	Pancreatic Cancer	(58)
Regulates fatty acid oxidation	Interacts with ω-3 polyunsaturated fatty acids	Prostate Cancer	(60)

### 5.2 Nitrogen balance and metabolic reprogramming

As a key enzyme in the glutamine-aspartate shuttle, GOT2 participates in nitrogen balance regulation and urea cycle metabolism. By directing the transfer of aspartate into the urea cycle, GOT2 promotes excretion and recycling of excess intracellular nitrogen, maintaining nitrogen metabolic homeostasis. Studies have shown that in colorectal cancer (CRC), the HIF-1α–SOX12 signaling axis activates GOT2 expression, driving asparagine biosynthesis, thereby simultaneously regulating the urea cycle and amino acid synthesis pathways. Specific inhibition of this signaling axis significantly reduces asparagine levels, thereby suppressing tumor cell proliferation through metabolic reprogramming (36). In hepatocellular carcinoma (HCC), Li et al. further demonstrated that GOT2 knockdown reprograms glutamine metabolism by enhancing glutaminolysis, nucleotide biosynthesis, and glutathione production, maintaining redox balance and promoting tumor progression via the PI3K/AKT/mTOR signaling pathway (37). Notably, the molecular mechanisms by which cancer cells dynamically allocate aspartate flux between the urea cycle and nucleotide synthesis pathways through GOT2 remain to be systematically elucidated.

### 5.3 Cellular senescence and antioxidant homeostasis

GOT2 has a dual role in regulating cellular senescence. During glutamine catabolism, it promotes aspartate production, dynamically modulating glutamate and glutathione synthesis, thereby maintaining ROS homeostasis and preventing lipid peroxidation damage. Abnormal GOT2 expression significantly alters the cellular response to senescence signals, and the resulting disruption of redox homeostasis directly impacts tumor cell proliferation and survival. Yang et al. revealed that mitochondrial GOT2 participates in the regulation of cellular senescence in pancreatic ductal adenocarcinoma (PDAC) by finely controlling ROS levels. Further studies indicated that GOT2 knockdown increases ROS levels in PDAC cells, triggering p27-mediated cellular senescence, a protective effect potentially closely related to chemotherapy resistance (38).

### 5.4 Other potentially associated metabolic pathways

As a mitochondrial transaminase with broad substrate specificity, GOT2 catalyzes the transamination of cysteine sulfinate to generate 3-sulfinylpyruvate, which can subsequently decompose into pyruvate and sulfur dioxide. This reaction potentially links GOT2 to the biosynthesis of taurine and suggests a role in maintaining cellular sulfur metabolic homeostasis (39, 40). Previous studies have shown that aberrant GOT2 expression—such as decreased tissue levels or elevated serum levels—is closely associated with dysregulation of the sulfur oxidation pathway in certain patients with myocardial injury or cardiovascular diseases. This may involve mitochondrial sulfur compound metabolism and lead to oxidative stress and cellular dysfunction (41). Although current research on GOT2-related sulfur metabolism has primarily focused on non-cancerous diseases, whether a similar mechanism operates in tumor cells to affect taurine synthesis or modulate sulfur oxidation remains to be determined. Given the potential roles of taurine and sulfur dioxide in regulating apoptosis, redox balance, and the immune microenvironment, investigating GOT2-mediated sulfur metabolism may offer new insights into cancer metabolic reprogramming.

## 6 GOT2 and the tumor microenvironment

### 6.1 Expression and function across multiple cell types

The multicellular expression pattern and functional heterogeneity of GOT2 within the tumor ecosystem have been progressively elucidated. GOT2 is not only highly expressed in cancer cells but is also broadly expressed in immune cells (such as T cells and macrophages) and stromal cells (such as fibroblasts and endothelial cells) within the tumor microenvironment. For instance, in a spontaneous pancreatic cancer transgenic model (KPC mice), quantitative immunohistochemical analyses revealed that although GOT2 protein expression is markedly downregulated in tumor epithelial cells, cancer-associated fibroblasts (CAFs) retain high levels of GOT2 expression, suggesting a compensatory role in stromal remodeling (12). Single-cell transcriptomic analysis further confirmed that GOT2 is widely expressed in non-malignant cells within the pancreatic tumor microenvironment, including macrophages, CD8^+^ T cells, and endothelial cells, with expression levels positively correlated with metabolic activity (42). Bioinformatic analyses also revealed broad GOT2 expression in immune and stromal compartments of clear cell renal cell carcinoma (43). Moreover, fourth-generation CAR-T cells engineered to stably overexpress GOT2 via lentiviral vectors demonstrated enhanced cytotoxicity against hepatocellular carcinoma cells in both *in vitro* and *in vivo* models (44).

### 6.2 Metabolic competition and immune microenvironment modulation

By catalyzing glutamine metabolism to produce aspartate and its derivatives, GOT2 not only maintains intracellular nitrogen metabolism and TCA cycle function but also supplies key metabolic substrates for immune cells. Aspartate is essential not only for pyrimidine and nucleotide biosynthesis but also plays an irreplaceable role in the activation, proliferation, and effector function of CD8^+^ T cells. Studies have shown that activated T cells are highly dependent on aspartate; insufficient aspartate availability leads to mitochondrial metabolic dysfunction, suppression of mTOR signaling, and reduced expression of cytotoxic molecules such as granzyme B (GZMB) and interferon-γ (IFN-γ), ultimately impairing T cell cytotoxicity (45). Tumor cells often upregulate GOT2 and associated metabolic pathways to enhance aspartate synthesis and uptake, thereby establishing metabolic competition with immune cells within the tumor microenvironment. Given the nutrient-restricted nature of the tumor microenvironment, preferential aspartate acquisition by tumor cells exacerbates its scarcity, severely limiting the metabolic adaptability and functional status of CD8^+^ T cells. This compromises their antitumor activity and facilitates immune evasion (46). Therefore, GOT2 not only participates in cancer cell metabolic reprogramming but also plays a pivotal role in shaping an immunosuppressive microenvironment.

### 6.3 Metabolic coupling with cancer-associated fibroblasts

CAFs support GOT2-mediated metabolic reactions by secreting nutrient substrates such as glutamine, thereby facilitating the production of energy and biosynthetic intermediates in tumor cells. In turn, aspartate or α-KG generated by tumor cell metabolism can regulate CAF metabolism, for example, by enhancing their lactate production capacity, establishing a bidirectional metabolic coupling. This cooperative interaction significantly enhances tumor cell proliferation and invasiveness (35, 47). A study by Kerk et al. further revealed this microenvironment-dependent metabolic complementation. In PDAC, while GOT2 knockdown significantly suppresses tumor cell proliferation *in vitro*, it does not impair tumor growth in xenograft or orthotopic mouse models. Mechanistically, CAFs—an essential component of the PDAC microenvironment—secrete redox-active pyruvate that provides alternative metabolic support to GOT2-deficient cells. When cultured in CAF-conditioned medium (CM), the proliferation of GOT2-deficient cells is partially restored. However, when pyruvate uptake is blocked or its conversion to lactate is inhibited, neither CAF CM nor exogenous pyruvate can rescue the proliferative capacity of GOT2-deficient cells (12). These findings underscore a functionally complementary metabolic interaction between tumor cells and CAFs, in which GOT2 serves as a critical mediator linking cancer and stromal metabolism.

### 6.4 Vesicle-mediated long-range effects and metastasis

In addition to interacting with neighboring cells in the local microenvironment through metabolic and signaling pathways, tumor cells can also engage in long-range communication via extracellular vesicles, particularly exosomes (48). Exosomes not only carry bioactive molecules such as miRNAs, proteins, and lipids, but may also encapsulate metabolic enzymes like GOT2 and their associated metabolites for delivery to adjacent or distant recipient cells. Through this mechanism, GOT2 may contribute to the formation of the pre-metastatic niche. For example, GOT2 transferred via exosomes to distant tissues may modulate the intracellular balance of glutamate and aspartate through its transamination activity, thereby influencing glutamine utilization, supporting aspartate biosynthesis, and promoting metabolic reprogramming to enhance the adaptability of recipient cells to new microenvironments. In metastatic lesions, this vesicle-mediated metabolic support mechanism facilitates cancer cell proliferation and survival, enhancing their competitive advantage and accelerating colonization and malignant progression at distant sites (49). Moreover, GOT2-associated metabolites may further modulate immune cell function and stromal remodeling within metastatic sites, contributing to the optimization of the metastatic microenvironment. Collectively, GOT2 not only maintains metabolic homeostasis within primary tumors but also functions as both a bridge and an amplifier of long-range metabolic regulation through exosome-mediated intercellular communication during metastasis.

## 7 Multi-level regulation of GOT2 in cancer

### 7.1 Transcriptional regulation

Various transcription factors dynamically regulate GOT2 transcription by binding to its promoter or enhancer regions. In breast cancer, the BRCA1/ZBRK1 complex represses GOT2 promoter activity, suggesting that BRCA1 loss-of-function may upregulate GOT2 by removing this repression (50). Notably, in diffuse large B-cell lymphoma, inflammation-related signaling pathways (e.g., STAT3 and NF-κB) indirectly enhance GOT2 transcription by upregulating c-Myc, thus meeting the high metabolic demands of tumor cells (51). Further studies revealed that SOX12 forms a metabolic regulatory network in CRC by activating glutaminase (GLS), GOT2, and asparagine synthetase (ASNS). Downregulation of GLS, GOT2, and ASNS effectively blocks SOX12-mediated CRC cell proliferation and metastasis, while ectopic expression of these enzymes reverses the inhibitory effects of SOX12 knockdown on tumor progression (36). Additionally, Stegen et al. identified a novel mechanism by which SOX9 regulates glutamine metabolism through c-MYC, simultaneously increasing expression levels of GLUD1, GLS1, and GOT2 (33).

### 7.2 DNA methylation regulation

GOT2 expression is dynamically regulated by epigenetic mechanisms, such as DNA methylation, but this regulation shows significant disease- and environment-dependent variability. In clear cell renal cell carcinoma (ccRCC), reduced GOT2 expression is directly associated with abnormally high methylation of its promoter region (43). Another study indicated that GOT2 expression in ccRCC patients is significantly correlated with methylation levels at multiple CpG sites, suggesting that epigenetic silencing of GOT2 may be a critical factor driving tumorigenesis (52). Notably, DNA methylation exerts opposite effects on GOT2 expression in different tumors. In pheochromocytoma, Remacha et al. found that high GOT2 expression correlates with abnormal accumulation of TCA cycle metabolites. Mechanistic studies demonstrated that mutations in TCA cycle-related genes in this tumor might alter DNA methyltransferase (DNMT) activity, inducing hypermethylation of promoter regions of multiple metabolic enzyme genes, including GOT2 (53).

### 7.3 Non-coding RNAs and post-transcriptional regulation

GOT2 mRNA levels are precisely regulated by a multi-layered network of non-coding RNAs. At the direct interaction level, Runtsch et al. found that ACOD1 lncRNA enhances GOT2 enzymatic activity by directly binding to the GOT2 protein, thereby driving tumor metabolic reprogramming (54). At the competitive regulation level, circRNAs function through a “molecular sponge” mechanism: Tang et al. demonstrated that Circ_0006220 in non-small cell lung cancer (NSCLC) stabilizes GOT2 mRNA and enhances its translation by sequestering miR-342-3p, thus promoting tumor angiogenesis and inhibiting apoptosis (55). Jin’s study showed that silencing circSEC31A releases miR-520a-5p, targeting and inhibiting GOT2, significantly reducing MAS function in NSCLC cells (56). Notably, clinical research has indicated the reversibility of this regulation—Prystupa et al. observed a dramatic decrease in GOT2 mRNA levels in peripheral blood leukocytes of NSCLC patients after radical surgery, highlighting the critical role of the tumor microenvironment in post-transcriptional regulation (57).

### 7.4 Post-translational modifications

GOT2 functions are dynamically regulated by post-translational modifications, including acetylation, phosphorylation, and succinylation, with modification patterns showing significant tumor type specificity. Among metabolism-enhancing modifications, acetylation has been identified as a critical regulatory mechanism. In pancreatic cancer, GOT2 acetylation promotes its interaction with malate dehydrogenase 2, forming a functional complex that activates MAS to maintain NADH/NAD^+^ homeostasis, thus driving tumor proliferation (58). In an inflammatory liver microenvironment, inflammation-induced GOT2 acetylation enhances its catalytic activity, promoting adaptive metabolic remodeling (59). Conversely, ω-3 polyunsaturated fatty acid-induced succinylation inhibits GOT2-mediated aspartate synthesis, disrupting essential metabolic pathways in prostate cancer cells and ultimately suppressing their proliferation (60). Notably, phosphorylation also participates in this regulatory network: activation-induced phosphorylation of GOT2 by protein kinase C epsilon significantly enhances its enzymatic activity, suggesting this modification acts as a key molecular switch regulating MAS functions (61). Although these findings underscore the importance of post-translational modifications in tumor metabolic reprogramming, the specificity and biological effects of these modification patterns across different tumor types still require systematic elucidation.

## 8 Potential and challenges of GOT2 as a theranostic target

By simultaneously regulating tumor metabolic reprogramming (maintaining aspartate supply) and redox homeostasis (regulating NAD^+^ regeneration), GOT2 has emerged as a promising anticancer target. However, clinical translation faces two major challenges (1): Target specificity—current inhibitors (e.g., amino acid analogs) struggle to distinguish between GOT1 and GOT2 isoenzymes; (2) Metabolic compensation risk—tumor cells may activate alternative pathways, such as pyruvate carboxylase, to compensate for GOT2 inhibition, leading to failure of single-target therapies (12, 62). Nevertheless, studies have proposed targeted interventions, including downregulating GOT2 via MYC/HIF-1α inhibition, developing allosteric inhibitors targeting the acetylation site K159 to induce tumor oxidative stress, and combining GOT2 inhibitor AOA with anti-GPC3 CAR-T therapy (such as BOXR1030) to enhance T-cell activity by depleting aspartate in the tumor microenvironment (36, 44, 58). Notably, the theranostic potential of GOT2 has expanded into dynamic monitoring. Combining plasma α-KG/aspartate ratio with ¹^8^F-FDG PET/CT imaging can establish a dynamic “metabolic biomarker-molecular imaging” system. This approach enables real-time monitoring of tumor metabolic activity (e.g., correlations between decreased glucose uptake and restored aspartate levels), achieving an integrated diagnostic-therapeutic loop. This development marks GOT2’s progression from basic research toward a unified “detection-intervention-monitoring” platform (37).

## 9 Conclusion and perspectives

As a recently identified “moonlighting enzyme,” GOT2 not only regulates classical metabolic pathways (e.g., the MAS-TCA cycle) but also profoundly influences the tumor metabolic-immune microenvironment through changes in subcellular localization and remodeling of protein interaction networks, exhibiting remarkable functional diversity and tumor-type specificity. Future studies should focus on developing precise GOT2-targeted drugs and combined metabolic-immunotherapy strategies, constructing multimodal platforms for dynamic diagnosis and treatment monitoring, and utilizing systems biology approaches to comprehensively understand the dynamic roles of GOT2 in the tumor microenvironment. These efforts will drive the transformation of cancer treatment from single-target interventions towards a novel therapeutic paradigm involving coordinated metabolic-immune network modulation.
